# Comparison of the Effects of the Alcalase-Hydrolysates of Caseinate, and of Fish and Bovine Gelatins on the Acidification and Textural Features of Set-Style Skimmed Yogurt-Type Products

**DOI:** 10.3390/foods8100501

**Published:** 2019-10-15

**Authors:** Yan-Shi Ma, Hui-Juan Zhao, Xin-Huai Zhao

**Affiliations:** Key Laboratory of Dairy Science, Ministry of Education, Northeast Agricultural University, Harbin 150030, Heilongjiang Province, China; mayanshi19900827@163.com (Y.-S.M.); zhaohuijuan915@126.com (H.-J.Z.)

**Keywords:** gelatin hydrolysate, casein hydrolysate, set-style yogurt, fermentation, texture

## Abstract

Commercial caseinate and two gelatins from bovine and fish skin were hydrolyzed by alcalase, and used at 2 g/kg in skimmed bovine milk that was then fermented with a commercial direct vat set starter, to clarify different effects of these hydrolysates on acidification and textural attributes of set-style yogurt samples. Compared with the fermentation of the yogurt sample without hydrolysate addition, the two gelatin hydrolysates in the yogurt samples endowed lower titratable acidity but higher pH values and thus delayed yogurt fermentation, while the caseinate hydrolysate showed an effect opposite to the two gelatin hydrolysates. The two gelatin hydrolysates induced worse quality attributes for the resultant yogurt samples, including higher syneresis extent, smaller hysteresis loop areas, and lower values in these textural indices like hardness, adhesiveness, apparent viscosity, elastic and viscous moduli. However, the caseinate hydrolysate led to improved quality attributes. Moreover, bovine gelatin hydrolysate always had a greater negative effect than fish gelatin hydrolysate on yogurt acidification and texture. It is concluded that these gelatin hydrolysates could confer the yogurt with intended bio-activities of gelatin hydrolysates but negatively impact yogurt acidification and texture, while the caseinate hydrolysate might be helpful for yogurt processing by shortening fermentation time and improving yogurt texture.

## 1. Introduction

In the last couple of decades, the growing demand for body health has increased the production of healthy and functional foods. Among these healthy and functional foods are fermented dairy products (especially yogurt) that offer several health benefits arisen from the fermentation of lactic acid bacteria. During the processing of yogurt products, food stabilizers can be used as yogurt additives for better quality attributes via improving consistency and micro-structure or decreasing syneresis of yogurt products [1,2]. In China, bovine gelatin is among these mostly applied additives in yogurt processing, because it demonstrates a powerful capacity to confer yogurt products (especially the set-style yogurt) with higher firmness and lower syneresis [3]. Other food additives or ingredients, such as prebiotics (e.g., inulin and β-glucan), probiotics (e.g., *Bifidobacterium bifidum* and *Lactobacillus acidophilus*), and several nutraceuticals (e.g., isoflavones and γ-aminobutyric acid), also can be added to yogurt products for an attractive dimension, aiming to reduce disease risk [4], prevent rotavirus gastroenteritis and cardiovascular disease [4,5], decrease blood pressure and gastrointestinal disorders [6], and bring about hypoglycemic effect [4].

Protein hydrolysates from various protein sources can be obtained through the catalyzing action of various proteases like trypsin, pepsin, and others [7], and are regarded to possess desired biofunctions such as anti-oxidant, anti-hypertensive, anti-bacterial, and immuno-modulatory effects [8,9]. For example, whey protein hydrolysate can prevent the oxidation initiated by free radicals and inhibit the activity of angiotensin-converting enzyme (ACE) [10]. It has been found that protein hydrolysates (such as casein hydrolysate) also were beneficial to yogurt processing, because their addition to yogurt milk could reduce the fermentation time of yogurt and enhance viable numbers of lactic acid bacteria [11,12]. According to the present knowledge, caseins (the main milk proteins) contain all of the amino acids necessary for the growth of lactic acid bacteria [13]. Therefore, casein hydrolysate is a potential ingredient for yogurt processing because it can promote the growth of yogurt starters and accelerate yogurt fermentation.

In several past studies, various gelatin hydrolysates were proved to have anti-oxidative [14], ACE-inhibitive [15], and more importantly, in vitro growth proliferation effect on osteoblastic cells by our research group [16,17,18]. Gelatin hydrolysates thus can be considered as possible healthy ingredients for the processing of functional foods. However, whether the application of gelatin hydrolysates in dairy products (e.g., yogurt or yogurt-like products) has a potential impact on dairy processing and product quality is unsolved in the present time, which might be an important issue to the yogurt producers. Based on this consideration, this study assessed the effects of two alcalase-generated gelatin hydrolysates (from bovine and fish skin gelatins) on the set-style skimmed yogurt samples using these indices like acid production, texture, and rheological properties as evaluation criteria, in comparison with these effects of the caseinate hydrolysate prepared with alcalase.

## 2. Materials and Methods

### 2.1. Materials

Commercial bovine and tilapia fish skin gelatins were bought from Shandong Yimin Biological Technology Co., Ltd. (Binzhou, Shandong, China), and measured with protein contents of 958 and 910 g/kg (dry basis), respectively. The caseinate was bought from Beijing Aoboxing Biotechnology Co., Ltd. (Beijing, China) with a protein content of 955 g/kg (dry basis). Alcalase (with a measured activity of 210 kU/g) was bought from Novozyme China (Tianjing, China). The skimmed bovine milk powder used in yogurt preparation was obtained from Fonterra Trading (Shanghai, China) Co., Ltd. (Shanghai, China), and determined negative in antibiotic residues. The commercial direct vat set (DVS) starter (YO-MIX 499) consisting of *Streptococcus thermophilus* and *Lactobacillus delbrueckii* subsp. *bulgaricus* was obtained from Danisco GmbH. (Beijing, China). The table sugar (i.e., commercial sucrose) was obtained from Dalian Minyipin Trading Co., Ltd. (Dalian, Liaoning, China). Other chemicals used were all of analytical grade. Distilled water was used in this study.

### 2.2. Preparation of Protein Hydrolysates

The two gelatin hydrolysates were prepared as previously described [16], while the caseinate hydrolysate was prepared using the methods described in a previous study [17]. In brief, the bovine and fish gelatins, as well as caseinate, were dispersed in water at fixed protein concentration of 50 g/kg and pH value of 8.0, and then hydrolyzed by alcalase (5, 6, and 2.5 kU/g protein) at 55 °C for 13, 13, and 3 h, respectively. The pH value of each hydrolysis mixture was adjusted by adding 1 mol/L NaOH at set intervals during hydrolysis. After that, these mixtures were heated for 15 min in a water bath operated at 95 °C to inactivate alcalase, centrifuged at 11,000× *g* for 20 min using a high speed freezing centrifuge (Type GL-21 M, Shanghai Centrifuge Institute Co., Ltd., Shanghai, China) to yield respective supernatants (i.e., protein hydrolysates), which were finally freeze-dried using an ALPHA 1–4 LSC plus freezes dryer (Marin Christ Co. Ltd., Osterode, Germany) and then stored at −20 °C before future use. In this study, the hydrolysates from bovine and fish gelatins as well as caseinate were assigned with respective abbreviation symbols BGH, TGH, and CH.

### 2.3. Preparation of Yogurt Samples

Set-style skimmed yogurt samples were prepared as follows. The skimmed milk powder was dissolved in water to obtain skimmed milk with protein concentration of 30 g/kg and pH value near 6.8 (using 0.2 mol/L NaOH). The skimmed milk was added to one of the three hydrolysates (2 g/kg) and table sugar (60 g/kg), and then was used to prepare the hydrolysate-added yogurt samples. The skimmed milk with table sugar addition but without hydrolysate addition was used to generate the control yogurt sample. All milk samples were then heated for 10 min at 90 °C, rapidly cooled to about 42 °C, mixed with the DVS starter at a level of 0.06 g/kg milk as recommended by the starter producer, sub-packed into the sterilized containers with 100 mL capacity under the aseptic conditions, covered with the sterilized poly-tetrafluoroethylene (PTFE) micro-pore membranes with gas permeability, and then fermented at 42 °C for 5 h. Afterwards, all yogurt samples were stored at 4 °C for 1 or 21 day, and then selected randomly for further assays. Moreover, some yogurt samples at various fermentation times (1, 2, 3, 4, and 5 h) were used to monitor their values of pH and titratable acidity.

To measure yogurt texture, yogurt samples of 80 mL were prepared in the beakers of 100 mL capacity. To measure yogurt syneresis, 20 mL yogurt milk was used to prepare yogurt samples in the centrifuge tubes of 50 mL capacity. These beakers and tubes were all sterilized at 121 °C for 20 min before their use and covered with the sterilized PTFE micro-pore membranes during yogurt fermentation. The prepared yogurt samples were all stored at 4 °C for 1 or 21 day, and then subjected for the measurements discussed below.

### 2.4. Chemical Analyses

Yogurt samples were analyzed for their protein, total solids contents, and titratable acidity (expressed as % lactic acid) using the Kjeldahl, gravimetric, and titration methods [19], respectively. A pH meter (Mettler, Toledo, DELTA-320 pH, Shanghai, China) was used to monitor the pH values of yogurt samples.

### 2.5. Assays of Yogurt Texture and Syneresis

Yogurt texture was measured using the combined compression and penetration test as previously described [20]. The Stable Micro Systems Texturometer (Model TA-XT2i, Stable Micro Systems Ltd, Surry, UK) with a 5 kg load cell was used in this assay. Before this assay, the yogurt samples that were kept in the containers (45 mm diameter × 80 mm height) were equilibrated to 20 °C. After that, a 35 mm diameter cylindrical rod (A/BE 35) was penetrated into the samples, using a constant cross-head velocity of 1 mm/s, a triggering force of 5.0 g, fixed sample depth of 30 mm, and two cycles. Four textural indices (hardness, adhesiveness, springiness, and cohesiveness) were thus obtained by using the XT.RA Dimension Ver. 3.7 software (Stable Micro Systems Ltd., Surry, UK).

Syneresis extent was assayed by a centrifugation method as previously described [21]. The yogurt samples in centrifuge tubes were centrifuged at 640× *g* for 10 min to separate their supernatants. The collected supernatants were weighed and used to calculate syneresis extent. The supernatant weights were expressed per initial sample weights of 100 g (as percentages).

### 2.6. Rheological Measurements

The assay of apparent viscosity was performed at 20 °C using a Bohlin Gemini II rheometer (Malvern Instruments Limited, Worcestershire, UK) equipped with a cone-plate geometry (diameter 40 mm, cone angle 4°, gap 0.15 mm), as the previous study did [22]. The yogurt samples were all initially equilibrated to 20 °C and then stirred softly for 17 times in clockwise direction using a spatula as an agitator to ensure they were in a uniform state before this assay. Apparent viscosity was quantified using shear rates of 0.1−10 s^−1^. Moreover, the elastic and viscous moduli (G’ and G’’) of the yogurt samples were also measured by the rheometer using the same cone-plate geometry, frequency sweeps of 0.1−10 Hz, and 0.5% strain, as the previous studies did [23,24].

Thixotropy was measured as previously described [24], using the rheometer and cone-plate geometry. The yogurt samples were loaded on the inset plate of the rheometer. A shear rate of 500 s^−1^ for 1 min was used to diminish potential structural differences among these yogurt samples. Flow curves of the yogurt samples were generated using shear rates from 0.1 to 100 s^−1^ (up and down sweeps). Hysteresis loop areas of the yogurt samples were thus calculated using the Rheo Win Pro software (Malvern Instruments Ltd., Worcestershire, UK).

### 2.7. Statistical Analysis

All experiments and analyses were carried out in triplicate (*n* = 3). The data were reported as means or means ± standard deviations. Significant differences were determined by the one-way analysis of variance (ANOVA) with Duncan’s test using the SPSS 13.0 software (SPSS Inc., Chicago, IL, USA). Differences were considered significant at *p* < 0.05.

## 3. Results and Discussion

### 3.1. Effects of the Added Hydrolysates on Acid Production of Yogurt Samples

The chemical assaying results showed that all prepared yogurt samples were similar in their protein (ranging from 27.6 to 30.2 g/kg) and total solids content (ranging from 145.2 to 147.7 g/kg). However, these yogurt samples showed some differences in their acid production during yogurt fermentation. In total, compared with the acid production in the control yogurt sample without hydrolysate addition, the addition of BGH and TGH in yogurt milk led to an inhibitory effect on acid production, while the addition of CH showed an opposite effect by enhancing acid production (Table 1). As a result, the control yogurt sample after 5-h fermentation had pH value of 4.56 or titratable acidity of 63.5, while the BGH- TGH-, and CH-added yogurt samples after 5-h fermentation had pH values of 4.83, 4.76, and 4.47 or titratable acidities of 50.0, 52.3, and 69.0, respectively (Table 1). The results illustrate that the addition of BGH and TGH in yogurt milk could delay yogurt fermentation, resulting in less acid production (or slower acidification). However, the addition of CH led to promoted yogurt fermentation, bringing about much acid production (or faster acidification). Compared with TGH, BGH mostly showed a similar inhibitory effect on acid production at the early fermentation stage (1−3 h), but exerted a greater inhibitory effect (*p* < 0.05) on acid production at the later fermentation stage (4−5 h).

Enzymatic hydrolysis causes no damage in amino acid compositions of proteins, but only leads to the breakage of the peptide bonds in the proteins. The amino acids or some peptides of lower molecular weights generated during this hydrolysis process can be used as nitrogen sources to support the growth of many bacteria [25,26]. Alcalase is an endoprotease with broad specificity for the peptide bonds of proteins [27], thus can be used efficiently in the preparation of various protein hydrolysates. Alcalase, therefore, was used in this study to generate the three hydrolysates from caseinate, fish, and bovine gelatins. It is known that lactic acid bacteria have a lower capacity to synthesize amino acids. Thereby, the growth of yogurt starters depends on exogenous nitrogen sources of amino acids and peptides. The availability of nitrogen sources (in the forms of proteins, peptides, or amino acids) is thereby considered essential for the growth of the starters [28]. Application of protein hydrolysates in yogurt milk thus can promote the growth of starter strains. It was evident that casein hydrolysates that contained mainly essential amino acids [29] could promote the growth of probiotic bacteria [30] and the strains of yogurt starters (i.e., S. thermophilus and L. bulgaricus) [31], and therefore resulted in reduced fermentation time [32]. The CH-added yogurt sample in this study thus obtained faster starter growth and had higher acid production or faster acidification than the control yogurt sample. However, gelatins are rich in these non-essential amino acids such as Gly, Pro, and especially 4-hydroxyl Pro [33]. The two gelatin hydrolysates in this study were also rich in these non-essential amino acids. Compared with the caseinate hydrolysate, the two gelatin hydrolysates thus had lower bioavailability than the starter strains. Consistent with the present results, it was also found that higher usage of the non-essential amino acids in the culture led to a decreased growth rate and lower biomass yield for *Lactococcus lactis* [34]. The addition of BGH and TGH in yogurt milk was accordingly observed to endow yogurt samples with higher pH values but lower titratable acidities (i.e., slower acidification). However, why BGH was more able than TGH to endow yogurt samples with higher pH values and lower acidification should be investigated in further study.

### 3.2. Effects of the Added Hydrolysates on Yogurt Texture and Syneresis Extent

The results (Table 2) show that these hydrolysates also had impact on textural features of the stored yogurt samples. In general, these yogurt samples stored at 4 °C for 21 days showed higher values in the four textural indices (hardness, adhesiveness, springiness, and cohesiveness) than those stored for 1 day. More importantly, both the BGH- and TGH-added yogurt samples always showed lower hardness and adhesiveness than the control yogurt sample of the same storage time. However, the CH-added yogurt sample always had higher values in hardness and adhesiveness than the control yogurt sample. Although the conducted BGH- and TGH- (or CH-) addition to yogurt milk brought about decreased (or increased) hardness and adhesiveness values (*p* < 0.05), these yogurt samples clearly showed close values in springiness and cohesiveness (*p* > 0.05). Compared with TGH, BGH always led to more decreases in the values of hardness and adhesiveness. Moreover, it was also found that the addition of BGH and TGH resulted in greater syneresis, as both the BGH- and TGH-added yogurt samples were detected to have higher syneresis extents than the control yogurt sample (41.2% and 37.3% versus 31.6% at 1 d, or 42.1% and 34.4% versus 33.2% at 21 days) (Table 3). Compared with BGH or TGH addition, CH addition showed a positive effect on yogurt quality, as this treatment led to the lowest syneresis values (28.4% or 29.8% at 1 or 21 days) for the resultant yogurt samples (*p* < 0.05) (Table 3). Higher syneresis extent reflected lower structural stabilization of the yogurt samples. TGH and especially BGH were thus regarded to have negative impact on yogurt texture and stabilization.

Adhesiveness is one of the important factors governing yogurt stability. A previous study had found that casein hydrolysate could improve yogurt texture and stability via increasing yogurt adhesiveness [30], which provided support to the present study. Hardness is another important factor to govern yogurt texture [35]. Moreover, there is a negative relationship between yogurt hardness and syneresis susceptibility [36,37]. Protein hydrolysates were considered to affect yogurt texture by changing the metabolism of starters and gel formation of milk proteins [38,39]. CH in this study led to much acid production (i.e., pH value near 4.5), while pH value near 4.5 is considered as a suitable condition for the caseins to form a gel network [40]. The CH-added yogurt sample was thus detected to have better texture (i.e., higher hardness and adhesiveness but lower syneresis extent) than the control yogurt samples. However, both BGH and TGH brought less acid production (i.e., pH values 4.7–4.8); subsequently, the BGH- and TGH-added yogurt samples received a looser gel network, and thereby had worse textural attributes, including decreased hardness and adhesiveness, but more enhanced syneresis extent than the CH-added yogurt sample.

### 3.3. Effects of the Added Hydrolysates on Apparent Viscosity and Yogurt Stabilization

**Figure 1:**
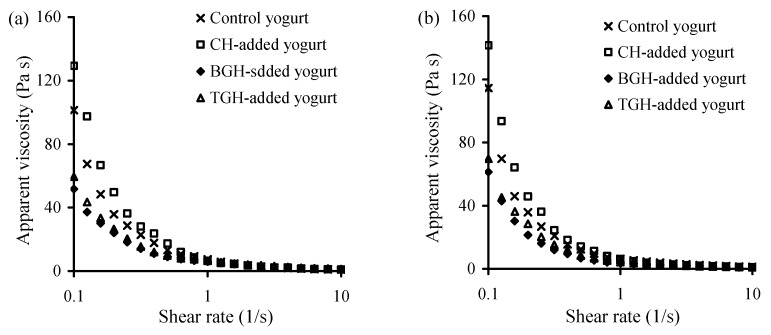
Apparent viscosity of the four yogurt samples stored at 4 °C for 1 (**a**) or 21 days (**b**).

Apparent viscosity of these yogurt samples at storage times of 1 and 21 days was evaluated to show possible changes in yogurt quality during yogurt storage. The results (Figure 1) indicated that the CH-added yogurt sample had the highest apparent viscosity in all cases, followed by the control yogurt sample, TGH-, and BGH-added yogurt samples. The addition of TGH and especially BGH to yogurt milk thus resulted in poor viscosity (i.e., thin) properties for the corresponding yogurt samples. BGH in usual resulted in the lowest apparent viscosity. Moreover, compared with those stored 1 d, the yogurt samples stored 21 days had value increases in apparent viscosity (Figure 1a versus Figure 1b). However, these TGH- and BGH-added yogurt samples at 21 days also showed lower viscosity values than the control or CH-added yogurt samples.

**Figure 2:**
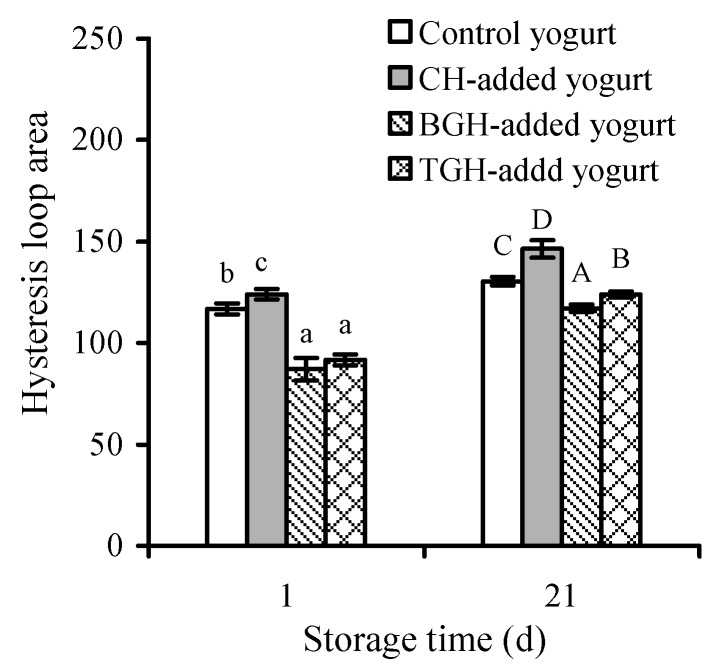
Measured thixotropy loop areas of the four yogurt samples stored at 4 °C for 1 or 21 days. Different capital or lowercase letters above the columns with the same storage time indicate that one-way ANOVA of the mean values differs significantly (*p* < 0.05).

The thixotropic analysis results showed that the addition of TGH and especially BGH in yogurt milk could confer the resultant yogurt samples with worse structural stabilization, reflected by their smaller hysteresis loop areas that are regarded to reflect required energy levels for structural breakdown and rebuilding (Figure 2). In detail, the TGH- and BGH-added yogurt samples gave hysteresis loop areas of 91.62 and 87.16 (1 day) or 123.9 and 117.1 (21 days), while the control yogurt sample showed a larger hysteresis loop area of 116.8 (1 day) or 130.4 (21 days). BGH, in general, led to the smallest hysteresis loop area. However, the CH-added yogurt sample had the highest values in hysteresis loop area (124.0−146.4), demonstrating that the CH-added yogurt sample had the highest structural stabilization. Moreover, the yogurt samples with storage time of 21 days also showed increased values in the hysteresis loop area, suggesting increased yogurt stabilization. These results proved again that the addition of TGH and BGH in yogurt milk had a negative effect on yogurt stabilization, showing a consistent conclusion with other assaying results (e.g., syneresis extents of yogurt samples) (Table 3).

Protein hydrolysates has been evident to influence the rheological properties of yogurt products. For example, casein hydrolysate prepared with papain was able to enhance yogurt viscosity [11]. The effect of protein hydrolysates on the apparent viscosity of yogurt may be related to their direct effect on milk acidification or acid production during yogurt fermentation [11,41]. Increased viscosity has been verified to ensure yogurt with better texture during low-temperature storage [39]. In this study, CH (but not BGH or TGH) addition exerted an accelerative effect on the acid production (Table 1), while only the CH-added yogurt sample had improved texture (Table 2 and Table 3). Thus, it was reasonable that the CH-added yogurt sample had higher values in apparent viscosity. Hysteresis loops are used to indicate the energy difference required for structural breakdown and rebuilding [42]. Stronger thixotropy yields a larger hysteresis loop area. Much energy thus is required for structural breakdown and rebuilding of the targeted samples, indicating higher structural stabilization. Unlike CH, both TGH and BGH led to smaller hysteresis loop areas, and thus endowed lower structural stabilization for the yogurt samples. Longer storage time ensured efficient interactions of these yogurt components. Thereby, the yogurt samples stored for 21 days showed increased apparent viscosity and stabilization than those stored for one day. Overall, it was evident that the addition of TGH and BGH to yogurt milk had a negative effect on yogurt stabilization. However, the addition of CH might improve yogurt stabilization.

### 3.4. Effects of the Added Hydrolysates on Elastic and Viscous Moduli

**Figure 3:**
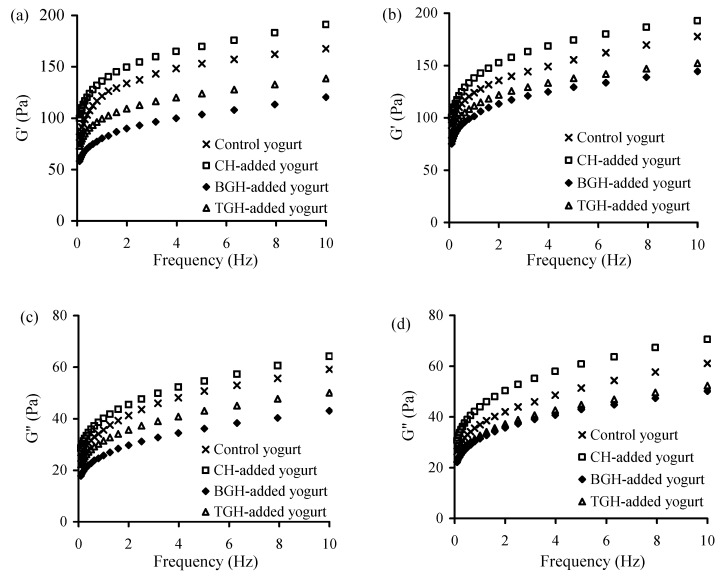
The measured G′ and G″ value of the four yogurt samples stored at 4 °C for 1 (**a**,**c**), or 21 days (**b**,**d**).

The elastic (G′) and viscous (G′′) moduli of these yogurt samples at storage times of 1 and 21 d were also evaluated using frequency sweeps of 0.1−10 Hz. The results (Figure 3) indicate that the CH-added yogurt sample had the highest G′ and G′′ values in all cases, followed by the control yogurt sample, TGH-, and BGH-added yogurt samples. The addition of BGH in general led to the lowest values for the two moduli. Moreover, long storage time consistently brought about increased moduli values (Figure 3a versus Figure 3b, or Figure 3c versus Figure 3d). It is, thus, concluded that the addition of TGH or BGH in yogurt milk led to reduced quality attributes of yogurt samples, but the addition of CH brought about improved quality attributes.

Viscoelasticity as one of the important physical indices of these semi-solid or viscous foods is reflected by elastic and viscous moduli, which thus serve as quality indices of these foods. Milk proteins, mainly caseins, under acidic conditions will form gels, the set-style yogurt, thus, behaves as a viscous or semi-solid matrix. To improve yogurt quality, milk proteins could be cross-linked by several enzymes to enhance protein interaction and to improve gel structure, for example, using transglutaminase or horseradish peroxidase in the presence of ferulic acid [43,44]. These treatments induced increased inter- and intra-interaction of milk proteins via various covalent bonds, and thereby, resulted in the acidified gels of milk proteins with increased G′ and G′′ values [43,44]. The treatment of yogurt milk by horseradish peroxidase, glucose oxidase, and glucose was also found to yield enhanced G′ and G′′ values for the yogurt samples, together with more compact and uniform microstructure [45]. Protein hydrolysates has been suggested to affect the G′ and G′′ values of yogurt, due to their direct effect on yogurt acidification [11,41]. Both TGH and BGH led to less acid production during yogurt fermentation. The caseins in yogurt milk in this circumstance had weaker interactions, and thus generated protein gels insufficiently in the resultant yogurt samples. It was reasonable that the TGH/BGH-added yogurt samples had looser and softer gel structure and were measured with lower G′ and G′′ values. In contrast to TGH and BGH, CH added in yogurt milk led to promoted acidification, which ensured a more compact gel structure. The CH-added yogurt sample thus had the highest G′ and G′′ values.

## 4. Conclusions

Based on the present assaying results, it was verified that the addition of the two alcalase-digested hydrolysates from the commercial bovine and fish gelatins at 2 g/kg milk could delay yogurt fermentation and lead to worse yogurt quality. In general, using the two gelatin hydrolysates was unfavorable as they might inhibit acid production during yogurt fermentation and more importantly, could bring about poor texture and stabilization for the yogurt samples that thus received lower values in textural indices (hardness and adhesiveness), apparent viscosity, elastic and viscous moduli, and hysteresis loop areas. Moreover, the alcalase-digested caseinate hydrolysate showed an opposite effect to the two gelatin hydrolysates, resulting in promoted acid production and improved texture and stabilization for the yogurt sample. It is concluded that although the two gelatin hydrolysates might confer yogurt products with those intended bio-activities of gelatin hydrolysates, their negative impacts on yogurt acidification and especially yogurt quality should be paid critical consideration. However, the caseinate hydrolysate might be a desired ingredient for yogurt processing by promoting yogurt acidification, shortening fermentation time, and improving yogurt texture.

## Figures and Tables

**Table 1:** The values of pH and titratable acidity of the four yogurt samples during a fermentation period of 1−5 h.

Index	Fermentation Time (h)	Control Yogurt	CH-Added Yogurt	BGH-Added Yogurt	TGH-Added Yogurt
pH	1	6.61 ± 0.01 ^a^	6.62 ± 0.01 ^a^	6.63 ± 0.01 ^a^	6.62 ± 0.01 ^a^
	2	6.12 ± 0.01 ^a^	6.15 ± 0.01 ^a^	6.35 ± 0.01 ^b^	6.33 ± 0.01 ^b^
	3	5.62 ± 0.01 ^a^	5.85 ± 0.01 ^b^	5.85 ± 0.01 ^b^	5.81 ± 0.01 ^b^
	4	4.86 ± 0.02 ^a^	4.82 ± 0.01 ^a^	5.25 ± 0.01 ^c^	5.14 ± 0.01 ^b^
	5	4.56 ± 0.01 ^b^	4.47 ± 0.01 ^a^	4.83 ± 0.02 ^d^	4.76 ± 0.01 ^c^
Titratable acidity	1	10.1 ± 0.6 ^A^	10.8 ± 0.8 ^AB^	11.8 ± 0.6 ^AB^	12.2 ± 0.1 ^B^
	2	10.9 ± 0.6 ^A^	12.0 ± 0.6 ^AB^	13.0 ± 0.6 ^BC^	13.7 ± 0.9 ^C^
	3	35.7 ± 0.6 ^B^	38.2 ± 0.8 ^C^	31.6 ± 0.1 ^A^	32.1 ± 0.4 ^A^
	4	55.1 ± 0.2 ^C^	58.0 ± 0.8 ^D^	46.9 ± 0.1 ^A^	48.6 ± 0.1 ^B^
	5	63.5 ± 0.8 ^C^	69.0 ± 0.8 ^D^	50.0 ± 0.4 ^A^	52.3 ± 0.5 ^B^

CH, BGH, and TGH represent caseinate, bovine gelatin, and fish gelatin hydrolysates, respectively. Different lowercase (or capital) letters after the data in the same row indicate that one-way ANOVA of the mean values differs significantly (*p* < 0.05).

**Table 2:** Textural indices of the four yogurt samples stored at 4 °C for 1 or 21 days.

Sample	Storage Time (days)	Hardness (g)	Adhesiveness (g s)	Springiness	Cohesiveness
Control yogurt	1	135.4 ± 2.7 ^c^	812.9 ± 12.5 ^c^	0.896 ± 0.002 ^a^	0.307 ± 0.013 ^ab^
	21	142.7 ± 1.0 ^C^	752.7 ± 17.2 ^C^	0.902 ± 0.009 ^A^	0.283 ± 0.010 ^A^
CH-added yogurt	1	159.3 ± 2.9 ^d^	855.8 ± 23.6 ^c^	0.901 ± 0.008 ^a^	0.290 ± 0.010 ^a^
	21	164.6 ± 4.8 ^D^	795.6 ± 16.6 ^C^	0.908 ± 0.018 ^A^	0.300 ± 0.010 ^A^
BGH-added yogurt	1	107.5 ± 2.1 ^a^	550.4 ± 7.5 ^a^	0.929 ± 0.019 ^a^	0.319 ± 0.012 ^ab^
	21	117.5 ± 1.4 ^A^	512.1 ± 12.9 ^A^	0.922 ± 0.012 ^A^	0.286 ± 0.010 ^A^
TGH-added yogurt	1	115.1 ± 4.0 ^b^	690.5 ± 9.4 ^b^	0.918 ± 0.016 ^a^	0.305 ± 0.010 ^ab^
	21	123.7 ± 1.5 ^B^	679.1 ± 33.5 ^B^	0.925 ± 0.008 ^A^	0.299 ± 0.008 ^A^

CH, BGH, and TGH represent caseinate, bovine gelatin, and fish gelatin hydrolysates, respectively. Different lowercase (or capital) letters after the data in the same column indicate that one-way ANOVA of the mean values at the storage time of 1 (or 21) day differs significantly (*p* < 0.05).

**Table 3:** The measured syneresis extents (%) of the four yogurt samples stored at 4 °C for 1 and 21 days.

Sample	Storage Time	
	1 day	21 days
Control yogurt	31.6 ± 0.5 ^b^	33.2 ± 0.6 ^B^
CH-added yogurt	28.4 ± 0.9 ^a^	29.8 ± 0.4 ^A^
BGH-added yogurt	41.2 ± 1.1 ^d^	42.1 ± 1.3 ^C^
TGH-added yogurt	37.3 ± 1.0 ^c^	34.4 ± 1.3 ^B^

CH, BGH, and TGH represent caseinate, bovine gelatin, and fish gelatin hydrolysates, respectively. Different lowercase (or capital) letters after the data in the same column indicate that one-way ANOVA of the mean values differs significantly (*p* < 0.05).
